# Selective targeting of microglia by quantum dots

**DOI:** 10.1186/1742-2094-9-22

**Published:** 2012-01-24

**Authors:** S Sakura Minami, Binggui Sun, Ketul Popat, Tiina Kauppinen, Mike Pleiss, Yungui Zhou, Michael E Ward, Paul Floreancig, Lennart Mucke, Tejal Desai, Li Gan

**Affiliations:** 1 Gladstone Institute of Neurological Disease, 1650 Owens St., San Francisco CA 94158, USA; 2 Department of Neurology, University of California, San Francisco, 505 Parnassus Ave., San Francisco CA, 94143, USA; 3 Department of Bioengineering and Therapeutic Sciences, University of California, San Francisco, 513 Parnassus Ave., San Francisco CA 94143, USA; 4 Department of Chemistry, University of Pittsburgh, 219 Parkman Ave., Pittsburgh PA 15260, USA; 5 Department of Mechanical Engineering, Colorado State University, Fort Collins CO 80523, USA

## Abstract

**Background:**

Microglia, the resident immune cells of the brain, have been implicated in brain injury and various neurological disorders. However, their precise roles in different pathophysiological situations remain enigmatic and may range from detrimental to protective. Targeting the delivery of biologically active compounds to microglia could help elucidate these roles and facilitate the therapeutic modulation of microglial functions in neurological diseases.

**Methods:**

Here we employ primary cell cultures and stereotaxic injections into mouse brain to investigate the cell type specific localization of semiconductor quantum dots (QDs) in vitro and in vivo. Two potential receptors for QDs are identified using pharmacological inhibitors and neutralizing antibodies.

**Results:**

In mixed primary cortical cultures, QDs were selectively taken up by microglia; this uptake was decreased by inhibitors of clathrin-dependent endocytosis, implicating the endosomal pathway as the major route of entry for QDs into microglia. Furthermore, inhibiting mannose receptors and macrophage scavenger receptors blocked the uptake of QDs by microglia, indicating that QD uptake occurs through microglia-specific receptor endocytosis. When injected into the brain, QDs were taken up primarily by microglia and with high efficiency. In primary cortical cultures, QDs conjugated to the toxin saporin depleted microglia in mixed primary cortical cultures, protecting neurons in these cultures against amyloid beta-induced neurotoxicity.

**Conclusions:**

These findings demonstrate that QDs can be used to specifically label and modulate microglia in primary cortical cultures and in brain and may allow for the selective delivery of therapeutic agents to these cells.

## Background

Chronic inflammation is a hallmark of many neurological diseases [1-5]. Microglia, innate immune cells of the CNS, become activated in response to injury and appear to have important roles in the defense against invading microbes and in wound repair [6]. They also phagocytose dead cells and help clear misfolded protein aggregates, such as those formed by amyloid beta (Aβ) in Alzheimer's disease (AD) [7]. However, under certain pathophysiological circumstances, microglia may also contribute to neuronal toxicity. For example, factors released from activated microglia can amplify inflammatory processes that contribute to neurodegeneration [8]. To harness and modulate the activity of microglia, it would be useful to be able to target biologically active compounds specifically to these powerful cells.

Previously, we used viral vectors and a microglia-specific promoter to selectively modulate gene expression in microglia [9]. However, the usefulness of this approach is limited by the possibility of inflammatory responses, potential toxicity associated with viral infections, and the inability of viral vectors to deliver a variety of chemical compounds. Here, we demonstrate that quantum dots (QDs) can effectively deliver biologically active molecules to microglia in vitro and in vivo.

Semiconductor fluorescent QDs are nanometer-sized particles with unique optical and electrical properties that make them particularly suited for visualization and tracking of living cells [10-12]. They have a heavy metal core, consisting for instance of cadmium and selenium or cadmium and tellurium, and an unreactive zinc sulfide shell. Manipulation of the core size allows synthesis of a wide array of QDs emitting at various wavelengths, visible as different colors. Because of their composition and small size, these nanoparticles are readily excitable by light and display minimal photobleaching [13]. Importantly, the outer coating can be modified to allow for the attachment of different bioactive molecules, offering unprecedented possibilities to visualize and modulate molecular processes in living cells [14,15]. QDs have been used for molecular imaging in diverse biological systems [16]. In most cases, surface-immobilized antibodies or peptides were used to direct QDs to specific cellular targets. For example, QDs conjugated to nerve growth factor (NGF) effectively activate TrkA receptors and downstream signaling cascades that promote neuronal differentiation [17]. QDs not conjugated to specific antibodies or peptides appear to have limited ability to enter most cells, especially at low concentrations [18]. Unconjugated QDs were found to be localized to macrophages and microglia that infiltrate experimental gliomas [19]. However, whether QDs are selectively taken up by microglia under normal conditions is unknown. Here we examined the ability of QDs to enter microglia in primary cultures and mouse brains and the underlying cellular mechanisms.

## Methods

### Quantification of QD uptake

Water-soluble ZnS capped CdSe streptavidin coated quantum dots (QDs) with emission at 655 nm were purchased from Invitrogen. QD solution was added to mixed cortical cultures at 0.5 nM for 1-48 h. The uptake of QDs was visualized under epifluorescence or confocal microscopy with an XF02-2 filter from Omega Optical that allows simultaneous multi-color viewing (Omega Optical, Brattleboro, VT). For visualization of QD655 uptake in mouse brain, confocal images were taken with a Nipkow spinning disk confocal microscope. GFP signal was imaged with a 488 nm laser and 515 nm bandpass, and QDs were imaged with a 405 nm and a 700 nm bandpass emission filter. Images were acquired in 0.5 nm step sizes in the z dimension. The amounts of QDs taken up by the cells were quantified with MetaMorph (Molecular Devices Corporation, Sunnyvale, CA). To investigate the mechanisms by which microglia take up QDs, cortical cultures were pretreated with chlorpromazine (CPZ) (6-20 μM), cytochalasin B (CTB) (4-8 μM), bafilomycin (BAF) (25-50 nM), mannan (1-2 mg/ml), polyinosinic acid (100-200 μg/ml), or blocking antibodies (anti-mannose receptor, anti-macrophage scavenger receptor, 2 μM) for 2 h before adding QD solutions, followed by 24 h incubation before analyses.

### Primary mixed culture and microglial culture

Cortices were isolated from Sprague-Dawley rat pups (Charles River Laboratories, Wilmington, MA) on postnatal day 0 or 1. To establish mixed cortical cultures, cells were plated at 160,000 cells/ml in plating medium containing Dulbecco's modified Eagle's medium (DMEM), 10% fetal bovine serum, 0.5 mM glutamax, and 100 U/ml penicillin and 100 μg/ml streptomycin for 7 days, as described [9].

Primary microglial cultures were prepared from 1-day-old mice as described [20]. Cortices were dissociated by mincing and incubation in papain and DNase. After centrifugation for 5 min at 500 × *g*, the cells were resuspended by trituration with a fire-polished Pasteur pipette, plated on 6-well plates (Falcon/BD Biosciences) at a density of 6.4 × 10^5 ^cells/well, and maintained in a 37°C, 5% CO_2 _incubator. The culture medium consisted of Eagle's minimal essential medium (MEM, Gibco) supplemented with 10% endotoxin-free fetal bovine serum (HyClone), 2 mM glutamine, and streptomycin. After 2 weeks in vitro, microglia were harvested by mildly shaking the cultures and collecting the floating cells. These cells were re-plated at a density of 5 × 10^5 ^cells/well in 24-well plates. The microglial cultures were used for experiments 2 days after re-plating. Each culture well was visually inspected by phase contrast microscopy before use, and wells containing contaminating astrocytes or greater than 30% activated microglia were excluded. Microglia with enlarged soma and less than two branching processes were considered activated. Experiments were performed in MEM, in which all drug stocks were diluted. All tissue culture supplies were purchased from Invitrogen (Carlsbad, CA) unless stated otherwise.

### Cytokine assay

Microglial cultures were placed in 300 μl MEM alone (ctrl) or with QDs (0.5 nM) for 6 h, after which the Qdots were washed out, and the microglia were placed in fresh MEM or lipopolysaccharide (LPS) (50 ng/ml) for 20-22 h. Medium samples were taken and treated with complete protease inhibitor and stored in -70°C. Medium (50 μl) was evaluated with a Beadlyte mouse 14-plex cytokine detection system (Millipore), according to the manufacturer's instructions. This immunoassay method employs 14 cytokine-specific antibodies tagged with fluorescent beads. Assays were performed in duplicate, and the fluorescent signal corresponding to each cytokine was measured with a BioPlex 200 system (Biorad). Values were normalized to the protein content of each well as determined by the bicinchoninic assay [21].

### Immunocytochemistry

Cultures were fixed in 4% paraformaldehyde in phosphate-buffered saline (PBS) for 15-30 min at room temperature. After permeabilization in PBS with 0.1% Triton for 10 min, cells were placed in blocking buffer (PBS with 10% FBS and 0.01% Triton) for 30 min. To label microglia, primary antibodies to Iba-1 (1:250, Wako Pure Chemical Industries, Osaka, Japan) or anti-CD11b (1:200, Chemicon, Temecula, CA) were applied in blocking buffer overnight at 4°C and visualized with anti-rabbit (Iba-1) or anti-rat (CD11b) conjugated with FITC (Vector Laboratories, Burlingame, CA). To identify astroglia or neurons, GFAP (1:1000; DAKO, Carpinteria, CA) or MAP2 (1:500; Chemicon, Temecula, CA) antibodies, respectively, were applied in blocking buffer for 2 h at room temperature or overnight at 4°C and visualized with anti-rabbit (GFAP) or anti-mouse (MAP2) conjugated with FITC (Vector Laboratories).

### Stereotaxic injection of QDs into the brain

The QD solution (3 μl at 100 nM) was stereotaxically injected into the hippocampus of *CX3CR^+/- ^*mice, which express green fluorescent protein (GFP) in microglia, at the following coordinates relative to bregma: anterior posterior: -2.1, medial lateral: ± 1.7, dorsal ventral: -2.0. The brains were perfused and fixed in 4% paraformaldehyde 2-28 days later.

### Quantum dots conjugation with saporin

The avidin-biotin affinity interaction was used to conjugate saporin to QDs. Briefly, 2 μl of QDs (1 μM) were mixed with 2 μl of biotinlyated saporin (56 μM), and 76 μl of PBS was added. The solutions were incubated at room temperature with continuous shaking for at least 2 h before they were used for analysis or treatment. X-ray photoelectron spectroscopy (XPS) analysis was performed to determine the chemical composition of saporin-conjugated QDs. Unconjugated and saporin-conjugated QDs were adsorbed on a silicon surface for ease with XPS analysis. An x-ray photoelectron spectrometer with a monochromatic Al-*Kα*-X-ray small spot source (1486.6 eV) and multichannel detector was used for this analysis. A concentric hemispherical analyzer was operated in constant analyzer transmission mode to measure the binding energies of emitted photoelectrons. The binding energy scale was calibrated by the Au4*f*7/2 peak at 83.9 eV, and the linearity was verified by the Cu3*p*1/2 and Cu2*p*3/2 peaks at 76.5 and 932.5 eV, respectively. Survey spectra were collected from 0 to 1100 eV with pass energy of 160 eV, and high-resolution spectra were collected for the C1*s *peak with pass energy of 10 eV. All spectra were referenced by setting the C1*s *peak to 285.2 eV to compensate for residual charging effects. Data for percent atomic composition and atomic ratios were calculated using analysis software. For peak fit analysis, a convolution of Gaussian components was assumed for all peak shapes. High-resolution C1s scans were taken to further support the presence of saporin on the QDs. The major hydrocarbon peak (C-C) is at 285.2 eV. The binding energy at 286.8 eV is assigned to amines (CH_2_N) and the binding energy at 288.0 eV is assigned to amide functional groups (O = C-N).

### Aβ treatment and cell death quantification

Aβ1-42 peptides lyophilized in hydroxyfluroisopropanol (HFIP) were purchased from rPeptide (Athens, GA). Lyophilized Aβ powder was reconstituted immediately before being diluted in Neurobasal A/N2 medium to 10-20 μM. Loss of neurons in mixed cultures was measured as described [9]. MAP2-positive neurons were counted in 15-40 random fields under a fluorescence microscope (400 × magnification).

## Results

### Selective uptake of QDs by microglia in primary cortical cultures

**Figure 1:**
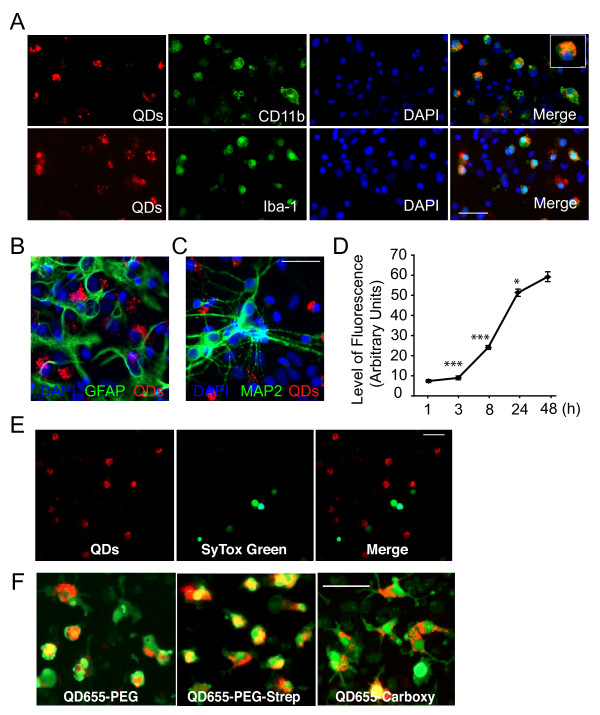
**Selective uptake of QDs by microglia in mixed primary cortical cultures**. **A**. In mixed primary cortical cultures from rats, QDs (red) were internalized primarily by microglia labeled with anti-CD11b (green) or anti-Iba-1 (green) antibodies. Nuclei were labeled with DAPI (blue). Scale bar, 50 μm. Inset, higher magnification of a QD-containing microglia cell labeled with Iba-1 antibody. **B, C**. QDs (red) were not found in astrocytes labeled with anti-GFAP (green, **B**) or in neurons labeled with anti-MAP2 (green, **C**). Scale bar, 50 μm. **D**. Fluorescence intensity of QDs in microglia increased with time of incubation. n = 30-100 cells from 5-6 independent images measured with Metamorph analyses. * *P *< 0.05 (24 h vs. 48 h), *** *P *< 0.001 (3 h vs. 8 h, and 8 h vs. 24 h); one-way ANOVA with Tukey-Kramer posthoc analyses. Error bars represent SEM. **E**. QDs (red) were not detected in apoptotic or necrotic cells labeled with Annexin V/Sytox green (green). Scale bar, 50 μm. **F**. Microglial internalization of QD655 conjugated with polyethylene glycol (QD655-PEG), PEG-streptavidin (QD655-PEG-Strep), or -COO^- ^(QD655-Carboxyl) in mixed cortical cultures. Red: QDs; green: Iba-1-positive microglia. Scale bar, 50 μm.

The passive uptake of QDs was investigated in primary cultures derived from neonatal rat cortices, which contain three main cell types: MAP2-positive neurons, GFAP-positive astrocytes, and CD11b-positive microglia [9]. Commercial QDs (QD-streptavidin, Invitrogen; emitting at 655 nm) were applied to the mixed culture system at different concentrations and visualized 1-48 h later. QDs were selectively internalized by microglia labeled with antibodies against CD11b or Iba-1 (Figure 1A). At QD concentrations of 0.5 nM, 38.9 ± 4.5% of Iba-1-positive microglia contained QDs after 18 h incubation. In contrast, no QDs were observed in GFAP-positive astroglia (Figure 1B) or MAP-2 positive neurons (Figure 1C). At 4 nM concentration, microglia started to internalize QDs within 1 h (Figure 1D), followed by a gradual intracellular accumulation of QDs during the next 48 h (Figure 1D). These results suggest that QDs are stable and not sensitive to intracellular degradation. Importantly, after 72 h incubation, QDs did not colocalize with Annexin V/Sytox green, which labels apopotic/necrotic cells (Figure 1E), suggesting that uptake and retention of QDs does not induce toxicity in culture.

To determine whether the selective uptake of QDs by microglia is influenced by surface chemistry, we used QDs with an amine-derivatized polyethylene glycol (PEG) outer coating that reacts directly with amine-reactive groups (QD655-PEG), QDs with a carboxyl coating (QD655-carboxy), or QDs with a PEG coating conjugated to streptavidin (QD655-PEG-Strep). QD655-PEG, QD655-PEG-Strep, and QD655-Carboxy were all selectively taken up by microglia in mixed cortical cultures (Figure 1F).

**Figure 2:**
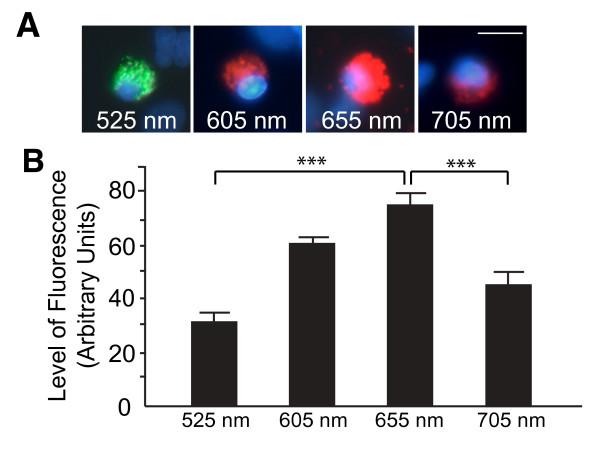
**Size of QDs affects their uptake by microglia**. **A**. Internalization of QDs of different sizes by microglia. Blue: DAPI. **B**. Size-dependent internalization of QDs by microglia was quantified by fluorescence intensity with Metamorph analyses. n = 23-39 cells from 5-6 independent images. ***, *P *< 0.001 by Tukey-Kramer posthoc test. Bars represent mean ± SEM. Scale bar, 20 μm.

We next tested whether the size of QDs affected their ability to enter microglia. QDs of different sizes emit at different wavelengths of the visible spectrum; therefore, QDs of different sizes can be distinguished by their color. We compared QDs emitting at 525, 605, 655, or 705 nm, and found that QD655 was the most efficient at entering microglia (Figure 2A-B). However, despite their different efficiencies, all QDs tested were selectively taken up by microglia in mixed cortical cultures, regardless of their emission wavelengths.

### QD uptake does not affect release of cytokines in primary microglia

To determine if uptake of QDs per se alters microglial function, we measured the release of inflammatory cytokines from primary microglia treated with QDs or with medium alone. QD treatment of primary microglia did not alter their release of tumor necrosis factor α (TNFα), KC, RANTES, MIP-1α, MIP-1β, or IP-10 (Table 1). Treatment with QDs also did not change the microglial release of cytokines in response to lipopolysaccharide (Table 1).

**Table 1:** Microglial release of cytokines

	TNFα	IL-1α	IL-1β	IL-6	KC	RANTES	G-CSF	MCP-1	MIP-1α	MIP-1β	IP-10
**Ctrl**	0.9 ± 0.5	ND	ND	2.3 ± 1.7	1.4 ± 0.8	2.3 ± 1.7	ND	ND	2.4 ± .9	3.8 ± 1.5	54.6 ± 35.3
											
(n = 3)											

**Qdots**	0.3 ± 0.1	ND	ND	ND	0.5 ± 0.1	1.2 ± 0.01	ND	ND	1.5 ± 1.0	3.2 ± 3.0	17.6 ± 9.8
											
(n = 2)											

**LPS**	226.5 ± 9.4	6.3 ± 3.2	1.7 ± 0.8	471.9 ± 34.6	345.5 ± 21.0	391.8 ± 19.0	53.9 ± 9.3	12.1 ± 7.2	592.7 ± 76.3	500.0 ± 23	189.8 ± 14.2
											
(n = 3)											

**LPS+**	217.1 ± 47.4	9.6 ± 6.9	2.6 ± 2.1	533 ± 197.5	363.5 ± 89.4	394.5 ± 84.2	49.4 ± 5.3	20.7 ± 17.9	637.3 ± 263.5	519.2 ± 169.0	196.0 ± 53.9
											
**Qdots **(n = 2)											

Cytokines (pg/ml) released by primary microglia treated with vehicle, QD, LPS, or LPS + QD (mean ± SEM). n, number of wells per condition. *ND *not detected, *G-CSF *granulocyte colony-stimulating factor, *IP-10 *10 kDa interferon-gamma-induced protein, *KC*, keratinocyte chemoattractant, *MCP-1 *monocyte chemotactic protein-1, *MIP-1α *macrophage inflammatory protein 1 alpha, *MIP-1β *macrophage inflammatory protein 1 beta, RANTES, regulated upon activation. The following cytokines were undetectable in all conditions: VEGF, IFNγ, and IL-10

### The uptake of QDs by microglia depends on clathrin-mediated endocytosis

**Figure 3:**
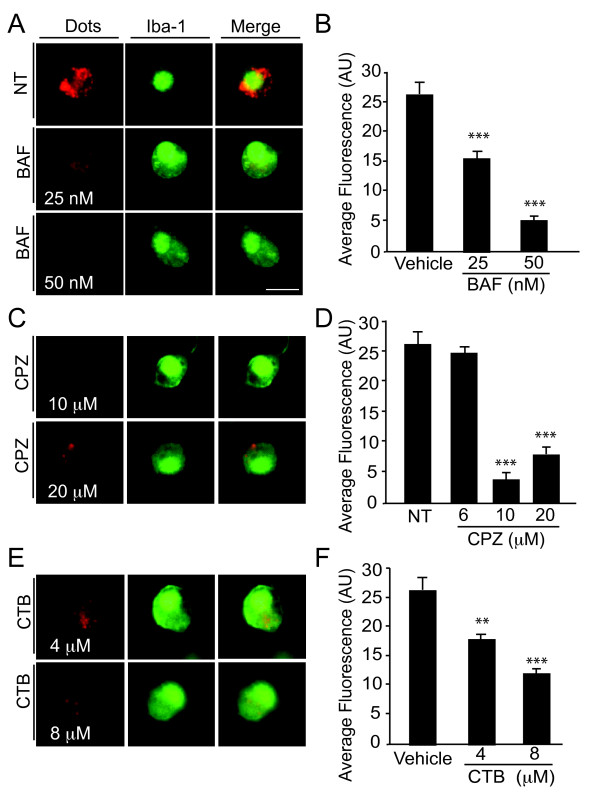
**The uptake of QDs by microglia depends on clathrin-mediated endocytosis**. The internalization of QDs by microglia was blocked dose-dependently by bafilomycin (BAF) (**A**, **B**), chlorpromazine (CPZ) (**C, D**), or cytochalasin B (CTB) (**E, F**). n = 18-48 cells per condition. ** *P *< 0.01, *** *P *< 0.001, vs. Vehicle (0.1% ethanol) or non-treated (NT) by Tukey-Kramer posthoc test. Bars represent mean ± SEM. Scale bar, 20 μm.

We next investigated the mechanism underlying the entry of QDs into microglia. Because the diameter of QDs ranges from 10-100 nm, we hypothesized that QDs traverse the cell membrane via endocytosis rather than phagocytosis [22]. Since pH sensitivity is considered a good indicator of entry by endocytosis, we examined if the uptake of QDs by microglia depends on pH. The specific inhibitor of endosomal proton-ATP pumps bafilomycin (BAF) elevates the pH in endocytic compartments to neutrality. The entry of QDs into microglia was blocked by BAF in a dose-dependent manner (Figure 3A-B), suggesting that this process depends on the acidification of endosomes.

To further dissect the molecular mechanisms underlying QD endocytosis, we investigated if the entry of QDs depends specifically on clathrin-mediated endocytosis, a well-studied mode of endocytosis that is crucial for many physiological functions, including the rapid clearance and down regulation of activated signaling receptors and the efficient recycling of synaptic vesicle membrane proteins after neurotransmission. Cells were treated with CPZ, a cationic amphiphilic drug that prevents the recycling of clathrin and thus prevents endocytosis by clathrin-dependent mechanisms [23]. Treatment with CPZ at 10-20 μM significantly inhibited the entry of QDs into microglia (Figure 3C-D). Clathrin-mediated entry can be also be inhibited by CTB, which induces depolymerization of actin filaments [24]. F-actin dynamics have been shown to be necessary for various stages of clathrin-coated vesicle formation, including coated pit formation, constriction, and internalization [25]. Treatment with CTB induced a dose-dependent decrease in QD entry into microglia (Figure 3E-F). Both CPZ and CTB inhibit transferrin (a specific maker for clathrin-mediated endocytosis) at the concentrations used in the current study [26], strongly supporting the notion that QDs enter microglia through clathrin-mediated endocytosis.

### The uptake of QDs by microglia occurs through mannose and macrophage scavenger receptors

**Figure 4:**
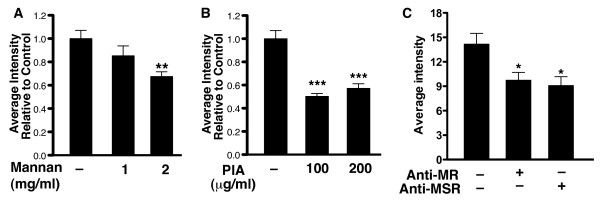
**Microglial uptake of QDs occurs through mannose receptors and macrophage scavenger receptors**. **A**. The internalization of QDs by microglia in rat primary mixed cortical cultures was quantified by fluorescence intensity with Metamorph software. Microglial uptake of QDs was blocked in a dose-dependent manner by mannan, an inhibitor of mannose receptors (**A**) and by polyinosinic acid (PIA), an inhibitor of scavenger receptors (**B**). n = 120-200 cells from at least eight separate images. Experiments were repeated three times. **C**. Antibodies against mannose receptor (MR) or macrophage scavenger receptor (MSR) were applied to rat primary mixed cortical cultures at a 2 μM concentration for 2 h before incubation with QDs. Microglial uptake of QDs was blocked by antibodies against MR and MSR. n = 105-120 cells from at least eight separate images. Experiments were repeated twice. ***P < 0.001, **P < 0.01, *P < 0.05 vs. control-treated, one-way ANOVA with Tukey-Kramer posthoc analyses. Bars represent mean ± SEM.

To determine if microglia-specific receptors, such as macrophage scavenger receptor 1 (MSR-1) [27-29] and mannose receptor [30], mediate the uptake of QDs, we treated primary mixed cortical cultures with inhibitors against these receptors and measured QD uptake by microglia. Mannan, an inhibitor of mannose receptor, dose-dependently decreased microglial uptake of QDs (Figure 4A). Polyinosinic acid (PIA), an inhibitor of macrophage scavenger receptor, also decreased microglial uptake of QDs (Figure 4B). Treatment with anti-mannose receptor (MR) or anti-macrophage scavenger receptor (MSR) antibodies also blocked microglial uptake of QDs (Figure 4C). These results implicate microglia-specific receptors in the selective uptake of QDs by microglia.

### Selective uptake of QDs by microglia in mouse brains

**Figure 5:**
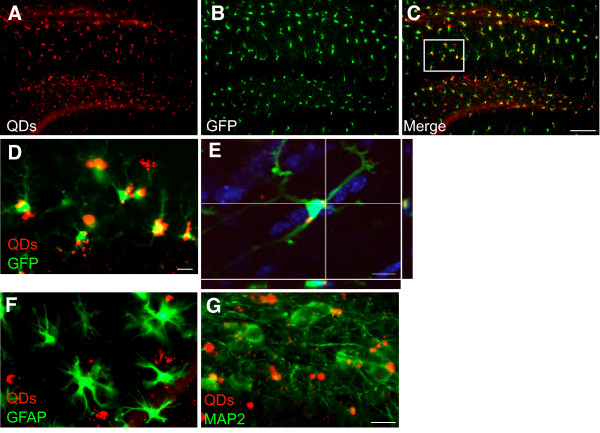
**QDs are selectively taken up by microglia in vivo**. **A-D**. QDs were stereotaxically injected into the hippocampus of *CX3CR*^+/- ^mice, which express GFP in microglia. **A**. Representative photomicrographs showing the distribution of QDs in the hippocampus. **B**. GFP-labeled microglia in the hippocampus. **C**. Merge of (**A**) and (**B**) shows that the majority of QDs were localized in microglia. Scale bar, 200 μm. **D**. Higher magnification image of the boxed area in (**C**). Scale bar, 20 μm. **E**. Representative confocal image through a single plane of quantum dots (red) internalized by microglia (green) in the hippocampus of *CX3CR*^+/- ^mice. Blue: DAPI. Scale bar, 20 μm. **F, G**. QDs (red) were not taken up by astrocytes labeled with anti-GFAP (green, **F**) or neurons labeled with anti-MAP2 (green, **G**) after they were injected into the hippocampus of C57Bl/6 wildtype mice. Scale bar, 20 μm.

**Figure 6:**
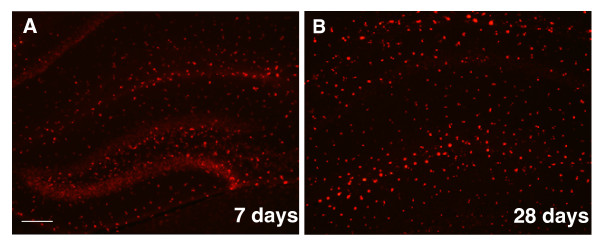
**Long-term expression of QDs**. **A, B**. QDs are observed 7 days (**A**) and 28 days (**B**) after injection into the hippocampus of adult C57BL/6 mice. Scale bar, 200 μm.

We next injected QDs (3 μl of 100 nM) into the hippocampus of *CX3CR^+/- ^*mice, which express green fluorescent protein (GFP) in microglia. QDs spread throughout most of the hippocampus (Figure 5A), Consistent with the selective targeting of QDs to microglia in cortical primary cultures, QDs were also predominantly localized in microglia in the brain (Figure 5C-D). Internalized QDs in microglia were further confirmed with Z-stack images (Figure 5E) and a 3D reconstruction of the confocal images (Additional file 1). In contrast, very little uptake of QDs was observed by GFAP-positive astroglia (Figure 5F) or MAP-2-positive neurons (Figure 5G). On some occasions, though, weak fluorescent signals were detected in the neurons of the dentate gyrus, suggesting limited neuronal uptake of QDs at high concentrations in vivo. Our results indicate that in the mouse brains, QDs target microglia preferentially and with high efficiency. Interestingly, the strong fluorescent signal remained stable for at least 1 month after the injection (Figure 6), supporting the feasibility of following the QDs long term.

### QD-saporin-mediated depletion of microglia decreases Aβ-induced neuronal loss

We next investigated if QDs could be used to deliver biologically active compounds selectively to microglia. Exposure of mixed cortical cultures to pathogenic Aβ aggregates, which are widely thought to cause AD, results in the degeneration of neurons. Notably, this neurotoxicity is at least partially dependent on the presence of microglia [31,32]. We therefore wanted to determine if the Aβ-induced neurotoxicity in such cultures could be suppressed by delivering a cytotoxin specifically to microglia through QDs. For this purpose, the cytotoxin saporin, which belongs to a family of single-chain ribosome inactivating proteins (RIPs), was biotinylated for coupling with QD-streptavidin. Saporin conjugated to an antibody against Mac1 (CD11b) has been used to selectively kill microglia in hippocampal slice cultures and in rat brain [33,34]. Here, we tested whether conjugation of saporin with QDs could result in similar microglia-specific cell loss.

**Figure 7:**
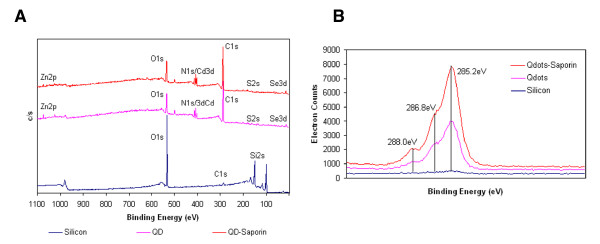
**Conjugation of QDs with saporin by streptavidin-biotin binding**. **A**. Comparison of carbon composition of QDs and QD-saporin conjugates measured by X-ray photoelectron spectroscopy. **B**. High-resolution C1 scans confirmed the presence of saporin on the surface of QDs.

To confirm the conjugation of saporin with QDs, XPS was used to compare the chemical composition of unconjugated and saporin-conjugated QDs adsorbed on a silicon surface. While negligible carbon was present on the silicon surface due to impurities, the carbon composition increased for the silicon surface adsorbed with QDs. There was a further increase in carbon for surface adsorbed with saporin-conjugated QDs, consistent with the presence of streptavidin and saporin, which are composed predominantly of carbon (Figure 7A, Table 2A). This increase was associated with a decrease in oxygen composition on the surfaces (Figure 7A, Table 2A). Moreover, there was a decrease in the elements present in QDs, namely Se, S, Cd and Zn after conjugation with saporin, further suggesting successful conjugation.

**Table 2:** Saporin conjugation of QDs characterized by X-ray photoelectron spectroscopy

		Binding Energy (eV)	Atomic %		
			
**A**.			Silicon	QDs	QD-Sap
	Se3d_3/2_	57	0	0.47	0.36
	
	Si2s	151	50.62	0	0
	
	S2p_1/2_	165	0	1.8	1.25
	
	C1s	285	9.09	78.08	80.27
	
	N1s	398	0	1.24	0.74
				
	Cd3d_5/2_	405			
	
	O1s	523	40.29	17.94	17.02
	
	Zn2p_3/2_	1022	0	0.47	0.36

B.			Silicon	QDs	QD-Sap

	285.2eV	C-H (hydrocarbon)	100	65	54
	
	286.8eV	CH_2_N (amine)	0	27	32
	
	288.0eV	O = C-N (amide)	0	8	14

**A**. Surface elemental composition calculated by XPS survey scans. **B**. High-resolution C1s peak deconvoluted into its components and indicating the composition of individual peaks on each surface

High-resolution C1s scans were taken to further support the presence of saporin on the QDs (Figure 5B, Table 2B). The major hydrocarbon peak (C-H) was at 285.2 eV. A binding energy at 286.8 eV is assigned to amines (CH_2_N), and a binding energy at 288.0 eV is assigned to amide functional groups (O = C-N). For the silicon surface, there was only one peak at 285.2 eV. For QDs, apart from the hydrocarbon peak at 285.2 eV, peaks for amine (at 286.8 eV) and amide (at 288 eV) were also present. After saporin conjugation, the intensities of the amine and amide peaks increased, as shown in the deconvolution of high-resolution C1s spectra into individual peaks. The decreased contribution of the hydrocarbon peak at 285.2 eV and the subsequent increase in amide and amine peaks at other binding energies reflect the successful conjugation of the QD surface with saporin.

**Figure 8:**
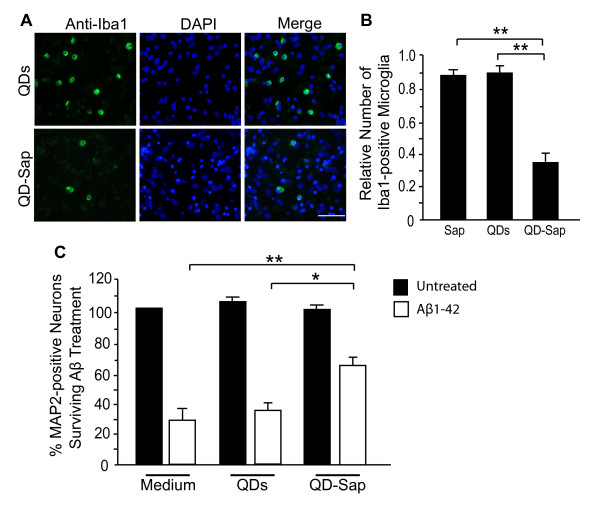
**QD-saporin-mediated depletion of microglia decreases neuronal loss in mixed cortical cultures exposed to Aβ**. **A**. Representative photomicrographs of microglial cells in mixed cortical cultures treated with QDs or QD-saporin (QD-Sap) conjugates. Microglial cells were labeled with anti-Iba-1 (green) and nuclei were labeled with DAPI (blue). **B**. Quantification of Iba-1-positive cells in mixed cultures treated with unconjugated saporin (Sap), QDs, or QD-saporin conjugates (QD-Sap). **C**. Quantification of MAP2-positive neurons in mixed cultures treated with Aβ1-42 after pretreatment with medium alone, QDs, or QD-Sap. n = 5 wells from three independent experiments. *P < 0.05, **P < 0.01, one-way ANOVA with Tukey-Kramer posthoc analyses. Scale bar, 100 μm.

We then applied QDs conjugated with saporin to primary cultures for 2 days and quantified microglia after the treatment by Iba-1 expression. Application of QD-Sap caused a marked reduction of microglia labeled with an antibody against Iba-1, without affecting the number or morphology of neurons or astroglia (Figure 8A and 8B). Treatment with unconjugated QDs or saporin alone did not significantly affect the number of microglia compared with untreated control (data not shown).

Next, we tested the effects of QD-Sap-induced microglial ablation on Aβ toxicity in mixed cortical cultures. Depletion of microglia with QD-Sap significantly increased the number of MAP2-positive neurons that survived Aβ1-42 treatment (Figure 8C). Application of QDs or saporin alone had no effects. Thus, neurons could be preserved in this neurodegenerative disease model through the selective targeting of microglia with modified QDs.

## Discussion

Our study shows that QDs are preferentially taken up by microglia in mixed cortical cultures and in brain. We further showed that the major cellular uptake pathway of QDs in microglia is clathrin-mediated endocytosis involving the microglia-specific receptors MSR-1 and mannose receptor. In primary mixed cortical cultures, QDs effectively delivered the cytotoxin saporin selectively to microglia. Depletion of microglia with QD-Sap resulted in protection against microglia-mediated Aβ toxicity.

Our finding that QDs are selectively taken up by microglia is consistent with previous observations that QDs were localized to macrophages and microglia that infiltrate experimental gliomas [19]. However, in contrast to the previous study, which suggested that QDs were phagocytosed by macrophages and microglia, our data indicate that QDs enter microglia via receptor binding and clathrin-mediated endocytosis. In eukaryotes, macromolecules enter the cell in membrane-bound vesicles either via 'phagocytosis' (the uptake of particles larger than 0.5 μm in diameter) or 'pinocytosis' (the uptake of fluid and solutes) [22]. Phagocytosis occurs by an actin-dependent mechanism and is usually independent of pH gradient and clathrin, whereas pinocytosis occurs by at least four basic mechanisms: macropinocytosis, clathrin-mediated endocytosis, calveolae-mediated endocytosis, and clathrin- and calveolae-independent endocytosis [22]. Interestingly, the uptake of soluble Aβ by microglia was found to be mediated through a nonsaturable, fluid phase macropinocytic mechanism that is distinct from phagocytosis and receptor-mediated endocytosis [35]. The size of QDs, which range from 10-100 nm in diameter, makes it unlikely that QDs enter microglia via phagocytosis. Indeed, the blockade of QD entry by balifomycin, chlorpromazine, and cytochalasin B provide strong evidence that QDs are taken up by microglia via clathrin-mediated endocytosis. Clathrin-mediated endocytosis occurs in all cell types. However, blocking the MSR-1 or mannose receptor with specific inhibitors or antibodies prevented the uptake of QDs, indicating that the selective targeting of QDs to microglia requires their binding to microglia-specific receptors. We cannot exclude that other microglial receptors such as Fc-receptors, complement receptors, and Toll-like receptors might also mediate the endocytosis of QDs by microglia. Additional studies are needed to fully characterize the potential binding sites of QDs on the microglial surface.

The unique optical properties of quantum dots, such as high quantum yields, large molar extinction coefficients, size-dependent tunable emission and high photostability, make them appealing as fluorescent probes for biological imaging. On the other hand, because of their size range, QDs are also very suitable for manipulations at the molecular level, offering new approaches for the delivery of potent bioactive agents. Microglia may have roles in the pathogenesis of various CNS diseases, including multiple sclerosis, Alzheimer's disease, Parkinson's disease, and amyotropic lateral sclerosis [36-39]. Our finding that certain sizes of QDs selectively target microglia provides a novel platform to probe and modulate biological processes in microglia and may lay the foundation for the development of QD-based reagents that can modulate specific signaling pathways in microglia.

In contemplating the therapeutic potential of QDs, an important caveat is their biocompatibility and toxicity [18,40]. The use of PEG on the surface of the QDs significantly improved their biocompatibility and minimized their toxicity [41-43]. A gene profiling study showed that application of high-dose QDs only induced changes in a small number of genes associated with the transport machinery, supporting the feasibility of long-term usage of QDs in biological systems [41]. The current study provides evidence that targeting of microglia with QDs is unlikely to result in toxicity through increased cytokine release, even in the presence of LPS-stimulated microglial activation. Indeed, our data suggest that the toxicity of QDs is limited, at least in the short term. However, evidence suggests that QDs may activate autophagy, implicating an important role in the regulation of normal cell processes [44-47]. The size-dependent induction of autophagy by QDs could result in the initiation of a cell death cascade [48]. Alternatively, the induction of autophagy during inflammation may protect against the harmful effects of microglial activation. Further investigation will be required to establish the long-term effects of the material, especially the heavy-metal component, in biological systems. If their safety profile continues to improve, QDs may emerge as a novel approach for the selective delivery of therapeutic agents to microglia in diverse CNS diseases.

## Conclusions

In conclusion, our study demonstrates that QDs can be used to specifically label and modulate microglia in primary cortical cultures and in the brain. This specificity is in part due to the selective uptake by macrophage scavenger receptors and mannose receptors present on the surface of microglia. These findings may allow for the selective imaging and delivery of therapeutic agents to microglia in a wide range of neurological disease models and, ultimately, perhaps also in the corresponding human conditions.

## Abbreviations

AD: Alzheimer's disease; BAF: balifomycin; CNS: central nervous system; CPZ: chlorpromazine; CTB: cytochalasin B; G-CSF: granulocyte colony-stimulating factor; GFAP: glial fibrillary acidic protein; GFP: green fluorescent protein; IFNγ: interferon-gamma; IL-10: interleukin 10; IP-10: 10-kDa interferon-gamma-induced protein; KC: keratinocyte chemoattractant; LPS: lipopolysaccharide; MAP2: microtubule-associated protein 2; MCP-1: monocyte chemotactic protein-1; MIP-1α: macrophage inflammatory protein 1 alpha; MIP-1β: macrophage inflammatory protein 1 beta; MSR-1: macrophage scavenger receptor 1; NGF: nerve growth factor; PEG: polyethylene glycol; QD: quantum dot; RANTES: regulated upon activation: normal T-cell expressed: and secreted (also known as chemokine (C-C motif) ligand 5 (CCL5)); RIP: ribosome inactivating protein; VEGF: vascular endothelial growth factor; XPS: X-ray photoelectron spectroscopy.

## Competing interests

The authors declare that they have no competing interests.

## Authors' contributions

SSM, BS, TK, KP, MEW, and YZ conducted experiments. LG, LM, and TD conceived the project. LG, BS, MP and SSM designed experiments. LG and SSM wrote the manuscript. All authors have read and approved the final manuscript.

## Supplementary Material

Additional file 1 **Quantum dots are internalized by microglia**. *CX3CR^+/- ^*mice, which express GFP in microglia, were injected with QD655 into the hippocampus. QD were imaged 48 h after injection, and z-stack confocal images were compiled into a 3D movie. Red: QDs, Green: microglia. Click here for file
